# Development and Performance Evaluation of Photovoltaic (PV) Evaluation and Fault Detection System Using Hardware-in-the-Loop Simulation for PV Applications

**DOI:** 10.3390/mi14030674

**Published:** 2023-03-18

**Authors:** Phuong-Truong Le, Huan-Liang Tsai, Phuong-Long Le

**Affiliations:** 1 Faculty of Mechatronics and Electronics, Lac Hong University, 10, Huynh Van Nghe St., Buu Long, Bien Hoa 810000, Dong Nai, Vietnam; 2 Department of Computer Science and Information Engineering, Da-Yeh University, Changhua 515006, Taiwan

**Keywords:** photovoltaic, PV evaluation and fault detection system, hardware-in-the-loop simulation

## Abstract

This paper originally presents a photovoltaic (PV) evaluation and fault detection (PVEFD) system for PV applications based on the Internet of Things (IoT) technology. The PVEFD system consists of an STM32F103C8T6 chip with a 32-bit Arm Cortex-M3 reduced instruction set computer (RISC) and 12-bit resolution analog-to-digital converter (ADC) to measure important parameters of PV applications, such as solar irradiance as well as the back-surface cell temperature, operating voltage, and output current of PV devices. The measured data of irradiance as well as back-surface cell temperature and operating voltage of PV devices are then fed into a built-in PV model in the on-chip Arm Cortex-M3 RISC for hardware-in-the-loop (HIL) simulation to obtain the simulated output current and power of PV devices. The resulting data are transmitted to a cloud server for remote monitoring and automatic warning function through a Raspberry PI 3 module and WiFi network. The simulation results are compared with in-field measurement data from PV modules and displayed on a human–machine interface (HMI) and an Android app. The results of the study illustrated that the proposed system features high accuracy and sufficient confidence. Furthermore, the fault detection function through the built-in HIL simulation function in PV systems was validated. Therefore, the proposed system is a small, compact, and cost-effective HIL-on-chip machine for remote surveillance of PV power systems.

## 1. Introduction

Photovoltaic electricity from solar energy has become a popular source of safe, sustainable, and efficient energy that converts solar radiation into electrical power through PV devices. However, real-time fault detection and energy conservation effectiveness remain challenges in the energy management system. PV panels are being developed for commercialized applications such as PV farms and rooftop PV installations [1,2]. Rooftop PV installations are being explored as potential sources of electricity for homes, farms, and grid connections [3,4]. Both conversion efficiency evaluation and fault detection have become critical in PV systems as they detect potential energy losses in real-time and in-field applications. There have been many developments in determining the faults of PV modules, including current and voltage deviation analysis, statistical analysis, signal processing, power loss analysis, and artificial intelligence methods. Paredes-Parra et al. [5] proposed a wireless PV monitoring system that assessed weather effects by collecting measurement data from sensors such as temperature, wind speed, humidity, and solar radiation. The data were recorded and transmitted in real-time to a remote control system in the cloud using a Raspberry Pi. Omana et al. [6] proposed a MOSFET on-state resistance method to protect PV systems. Their experiment detected inverter MOSFET value changes early via an alarm message, which can be used to improve inverter efficiency and prevent interruptions in the PV system. Chao et al. [7] proposed a fault diagnostic system for PV applications using a neural network and a wireless Zigbee module with a PIC controller, which shows accurate results and is of lighter weight than traditional systems. Abdullah and Rosd [8] used low-cost hardware including Arduino Uno R3 and ESP8266 WiFi modules to implement a solar energy monitoring platform. Didi and Azami [9] measured operating voltage and output current at each PV terminal using ESP32 and Bluetooth HC05 to illustrate the simplicity and affordability of adding a Bluetooth link to IoT technology. Pachauri et al. [10] developed a data collection system that continuously monitored current and voltage to detect the effect of shadow problems on PV performance, which showed reductions between 0.48% and 1.95%. On the other hand, the authors of several studies have proposed online monitoring systems for PV arrays based on various technologies. Li et al. [11] used a Raspberry Pi platform and an FFA-SVM-based diagnostic model to diagnose faults in a PV array, which achieved a diagnostic accuracy of 98.5%. Reddy et al. [12] proposed a combination of wireless sensor networks (WSN) and IoT for smart farming. The results demonstrated that a smart farm node can predict power generation, reduce power consumption, and extend the lifetime of an energy harvest device. Ongaro et al. [13] proposed a hybrid energy storage system for a PV-based WSN system. Their results showed that combining energy storage strategies increased the lifetime of the battery system. Balakumar et al. [14] demonstrated a monitoring IOT system for PV devices using machine learning technology. Das et al. [15] studied the impact of sunlight on PV performance and suggested using optical wireless communication receivers to increase power harvest. Tavakkolnia et al. [16] studied simultaneous energy harvesting and high-speed MIMO optical wireless communication using organic PV devices. Habib et al. [17] proposed using IoT to monitor the grid status and weather data of a large photovoltaic plant. The authors suggested that faults in a 30-bus test grid model were caused by inverter power capacity and weather conditions. To address this, they proposed a continuous data transmission system using wireless communication. Their experiments showed that this method improved the voltage profile and reduced power losses by 24.1%. Monitoring PV plants and optimizing PV energy efficiency are critical issues for increasing electrical power demand. Therefore, Zhou et. al. [18] suggested a self-powered wireless charging system that charges electric automobiles by converting solar energy into alternating current (AC) electricity. Their research has broad applications for electric vehicles and offers advantages including clean, economical, and renewable energy. However, failures in PV arrays are frequently difficult to avoid and can result in energy loss, shutdowns, or even critical safety issues. Therefore, Corti et al. [19] proposed power management for smart farming wireless sensor networks. They investigated the control strategy for charging batteries via PV sources using Pulse Skipping Modulation (PSM) technology and promoted its energy efficiency. Their experimental results assumed that the converted energy efficiency from solar radiation can be used to avoid faults at different output power levels. A maximum power point tracking (MPPT) DC–DC converter using wireless power transmission was proposed by Zheng et al. [20]. Their experimental findings showed that adding the MPPT to an inverter resulted in a significant energy transfer to the load. Moreover, smart monitor systems for PV devices have been proposed recently. Pereira et al. [21] used low-cost Raspberry Pi hardware and free Python software to develop a PV monitor system in real time. They developed a website to monitor the effects of temperature, radiation, humidity, and the environment on the PV output power. They found that smart monitoring increased PV power.

Recently, developments in DAQ configuration for monitoring PV systems have been mentioned, including data collection, processing, transport, and storage. The database was kept on a computer, and the local PC was used by an instrument called an Arduino Uno. The Monitoring Plant Network is critical to the system because it monitors PV status and sends real-time data to the Monitoring Center system. The Monitoring Plant Network is based on the concept of “smart” PV modules, in which sensors and electronic components are configured to control each panel or group of panels. These Smart Modules can acquire voltage, current, and temperature and send the data to a service center through a wireless network (Monitoring Center). Zhang et al. [22] used a WSN to monitor the performance of an organic PV system. Their configuration models combined various photovoltaic and battery sizes while employing machine learning algorithms to predict the effect of indoor and outdoor operation conditions. They concluded that energy savings indoors are possible at a high luminance level of 1000 Lux with a 10% relative drop in PV output power. Hu et al. [23] also looked at big data training utilizing WSNs to monitor PV power plants. Their findings showed that a prediction algorithm for PV output power may detect a PV sub-system fault in advance. Derdar et al. [24] proposed an Adaptive Network Fuzzy Inference System (ANFIS) versus an Artificial Neural Network (ANN)-based structure using a WSN to evaluate the energy efficiency of PV devices. First, a sensor node monitored the temperature and solar radiation. Then, all measurement data were recorded in order to calculate PV performance. The experimental results showed that the ANFIS model improved power point tracking ability and reduced energy loss. On the other hand, Xie et al. [25] investigated a PVM-oriented mismatch detection system using wireless sensing technology and achieved 100% accuracy in detecting array aging, shadow, and open circuit faults. Iqbal et al. [26] proposed a grid-connected PV plant with a rated power of 125 kWp to compare actual performance with theoretical results. They developed an algorithm utilizing a pyranometer and temperature sensors to predict the power output accuracy and detect the errors between the predicted and measured results of the PV plant. The study concluded that real-time weather monitoring and early fault detection in PV systems can improve accuracy. The use of wireless monitoring systems in PV fault detection has been explored by several researchers. Voutsinas et al. [27] proposed an ANN fault detection and identification for PV systems. The accuracy of fault detection reached up to 93.4%. Furthermore, Arduino Mega 2560 (ATmega2560), Arduino Uno, ESP8266, NodeMCU ESP8266, ESP32, and Raspberry Pi modules were used for both offline and online monitoring PV devices. Furthermore, Zakir et al. originally proposed a fault detection, localization, and categorization (FDLC) technology with a voltage sensor in a PV-fed DC-microgrid (DCMG) and experimentally validated its six statements [28]. This work illustrated the cost-effectiveness of the FDLC method only with a voltage sensor. Furthermore, the FDLC method for its five statements was evaluated by Zakir et al. using PSIM simulation in the MATLAB environment, which featured much flexibility in the simulation model [29]. These studies motivate us to propose an online monitoring system based on the STM32F407VGT6 chip and the Raspberry Pi 3 to collect cell temperature, solar radiation, operating voltage, and output current of PV devices to calculate the PV output power. The fault diagnostic prediction was built in based on the PV simulation model for commercialized PV devices. The emulation of a PV device with a well-developed PV model simulation using measured data of irradiance, cell temperature, and operating voltage of PV devices, and the comparison of measured and simulated results are carried out concurrently in an HMI and mobile phone.

A PVEFD system using a HIL simulation based on IoT technology is presented for developing and testing real-time systems for PV applications. A mathematical model of the PV system dynamics is created for the HIL simulation and a built-in simulator is integrated into the 32-bit Arm Cortex-M3 RISC in an STM32F103C8T6 chip. The PVEFD system is cost-effectively used to evaluate with the HIL simulator on the same platform. The remainder of this paper is organized as follows. First, the system design and the related PV theory are described in Section 2. Section 3 illustrates the design and implementation of the proposed wireless PVEFD system in the proposed wireless PV monitoring IoT system and visualization of solar irradiance as well back-surface cell temperature, operating voltage, output current, and power of PV devices on the cloud IoT platform. The in-situ evaluations and fault detection function are also illustrated in Section 4. Finally, brief conclusions and future works are outlined in Section 5.

## 2. Development of IoT-Based PVEFD System

**Figure 1:**
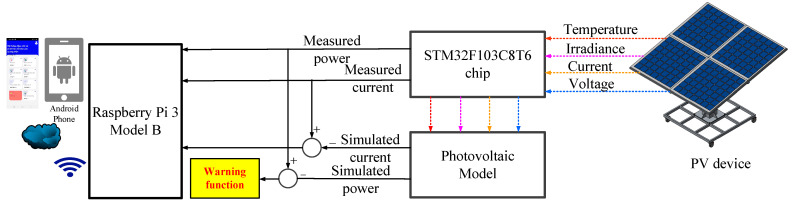
Configuration of proposed IoT-based PVEFD system.

Figure 1 shows the proposed IoT-based PVEFD system. The Arduino IDE V1 is used to acquire real-time data such as irradiance, cell temperature, voltage, and current from a commercial PV module. These data are then used to emulate the PV based on a well-developed PV model for commercial modules. The output current and power of the simulation are compared to the measured values from the PV device. Both simulation and measurement are integrated into the Arduino IDE platform for ease of use.

### 2.1. System Description

As shown in Figure 1, the hardware configuration of the proposed IoT-based PVEFD system includes a data acquisition device, an STM32F103C8T6 chip which consists of a 12-bit ADC, and a built-in PV model for HIL-based performance evaluation and fault detection, both of which are programmed using the Arduino IDE software. The hardware and software components are described in detail as follows.

### 2.2. PV Mathematical Model

It is well-known that PV conversion is a direct electromagnetic radiation conversion into electrical power. A solar cell consists of a p-n junction fabricated in a wafer or semiconductor. In general, the PV output power of a typical solar cell is less than 2 W at an operating voltage of 0.5 V. Solar cells are mounted in a commercial PV module in series configurations to produce the higher operating voltage and output power. For PV power applications, PV modules are arranged in series and/or parallel configurations in order to match the following power conditioning units (PCU) such as the DC/DC converter or DC/AC inverter. PV devices naturally exhibit nonlinear *I*-*V* and *P*-*V* characteristics that mainly depend upon solar irradiance and cell temperature. The PV cell temperature is a function of several variables, such as cell material, packing factor and thermal mass of the PV module, and ambient weather conditions. Based on the datasheet of PV manufacturers, the PV model can be built in the STM32F103C8T6 chip according to well-known formulas with sufficient precision and confidence for commercially available PV modules. In general, solar cells naturally exhibit nonlinear *I*-*V* and *P*-*V* characteristics that vary with solar radiation intensity and cell temperature. The *I*-*V* output characteristic equation of a PV device [30] is given by

(1) $$I=N_{P}I_{\mathrm{PH}}-N_{P}I_{S}\left\{\exp\left[\frac{q}{kT_{\mathrm{PV}}A}\left(\frac{V}{N_{S}}+\frac{IR_{S}}{N_{P}}\right)\right]-1\right\}-\frac{1}{R_{\mathrm{SH}}}\left(\frac{N_{P}V}{N_{S}}+IR_{S}\right)$$

where $N_{P}$ and $N_{S}$ are the numbers of solar cells in parallel and in series in a PV device, $I_{\mathrm{PH}}$ is the photocurrent, $I_{S}$ is the cell saturation of dark current, *q* is the charge of an electron, *k* is the Boltzmann’s constant, $T_{\mathrm{PV}}$ is the cell temperature that is assumed to be uniform in the PV module, *A* is the ideal factor that depends on PV technology, $R_{\mathrm{SH}}$ and $R_{S}$ are the resistance of shunt and series resistors, and exp(⋅) is the exponential function. The photocurrent mainly depends on the solar irradiance and the cell’s working temperature and is obtained by

(2) $$I_{\mathrm{PH}}=\left[I_{\mathrm{SC}}^{\mathrm{STC}}+K_{I}\left(T_{\mathrm{PV}}-T_{\mathrm{PV}}^{\mathrm{STC}}\right)\right]\frac{G}{G^{\mathrm{STC}}}$$

where $I_{\mathrm{SC}}^{\mathrm{STC}}$ is the short-circuit current of the PV module at the standard test condition (STC) of $T_{\mathrm{PV}}^{\mathrm{STC}}=25{\;}^{\circ }C$ and $G^{\mathrm{STC}}=1{\;\mathrm{kW}/m}^{2}$, and $K_{I}$ is the temperature coefficient of the cell’s short-circuit current. In addition, the cell’s saturation current varies with the cell temperature and is described as

(3) $$I_{S}=I_{\mathrm{RS}}{\left(\frac{T_{\mathrm{PV}}}{T_{\mathrm{PV}}^{\mathrm{STC}}}\right)}^{3}\exp\left[\frac{qE_{\mathrm{BG}}}{kA}\left(\frac{1}{T_{\mathrm{PV}}^{\mathrm{STC}}}-\frac{1}{T_{\mathrm{PV}}}\right)\right]$$

where $I_{\mathrm{RS}}$ is the reverse saturation current of the solar cell, and $E_{\mathrm{BG}}$ is the band-gap energy of the semiconductor used in the solar cell. Having an operating voltage *V*, the PV output power is directly calculated by

(4) $$P_{\mathrm{PV}}=IV$$

### 2.3. Firmware Design

**Figure 2:**
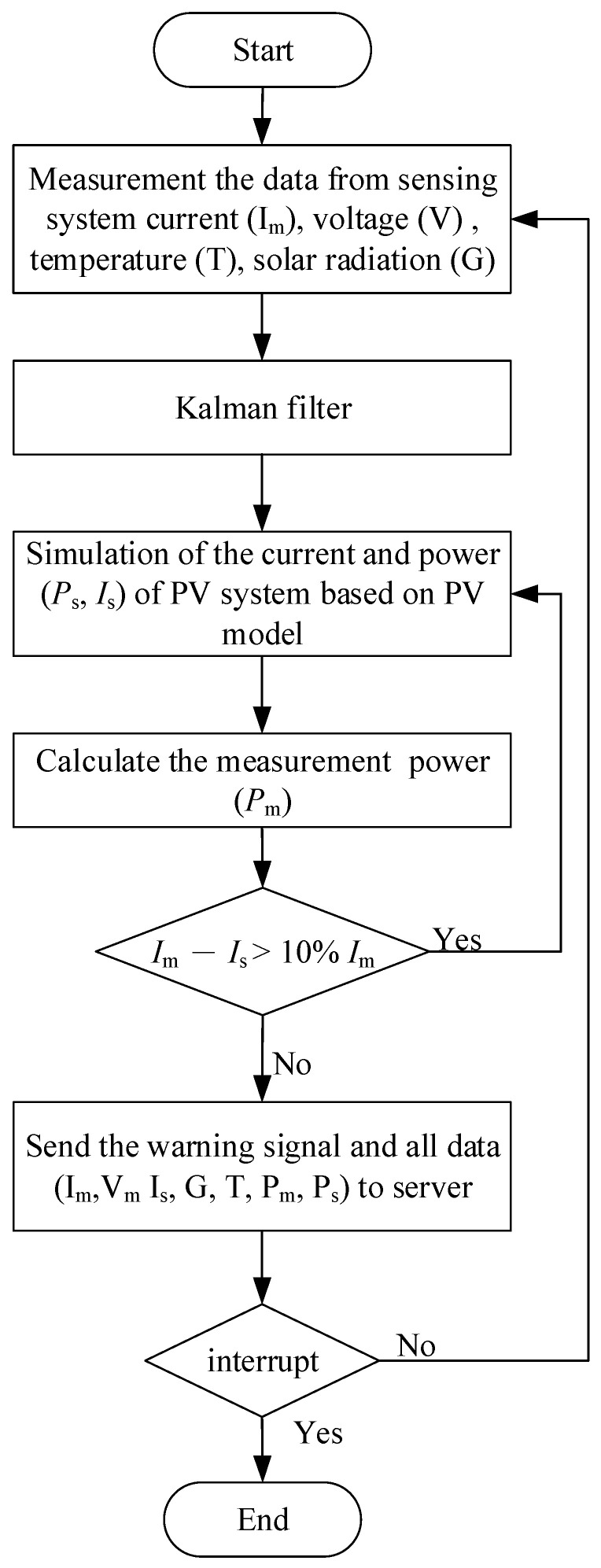
Flowchart of proposed IoT-based PVEFD system.

From the IoT viewpoint, the observation of solar irradiance by a pyranometer as well as the temperature, voltage, and current sensor for PV devices, can be referred to as the “physical layer” or “sensing layer”. In addition, the STM32F103C8T6 chip with a built-in Arm cortex-M3 system-on-chip (SoC) used for both HIL simulation and power calculation can be regarded as a “physical layer”. The Raspberry Pi model 3 with a WiFi module receives data from the main board STM32F103C8T6 via UART Tx/Rx interface, and all data are collected and displayed through the Raspberry Pi 3 module, and then forwarded to the “information layer” by the host server in the cloud through a WiFi access point (AP). The algorithm flowchart of the system is presented as shown in Figure 2. First, the sensing data of the irradiance, cell temperature, operating voltage, and output current of the PV devices are collected and sent to the 12-bit ADC of the STM32F103C8T6 chip. The built-in Arm cortex-M3 SoC will calculate the simulated current and power corresponding to the input data of the sensor based on the above mathematical model of the PV devices according to the Equations (1)–(4), the Raspberry Pi 3 module receives data from the main board STM32F103C8T6 via the UART Tx/Rx (Pins 8 and 10) interface, and all data collected and calculated will be sent to the host and displayed through Raspberry Pi 3 module.

### 2.4. Hardware Design 

**Figure 3:**
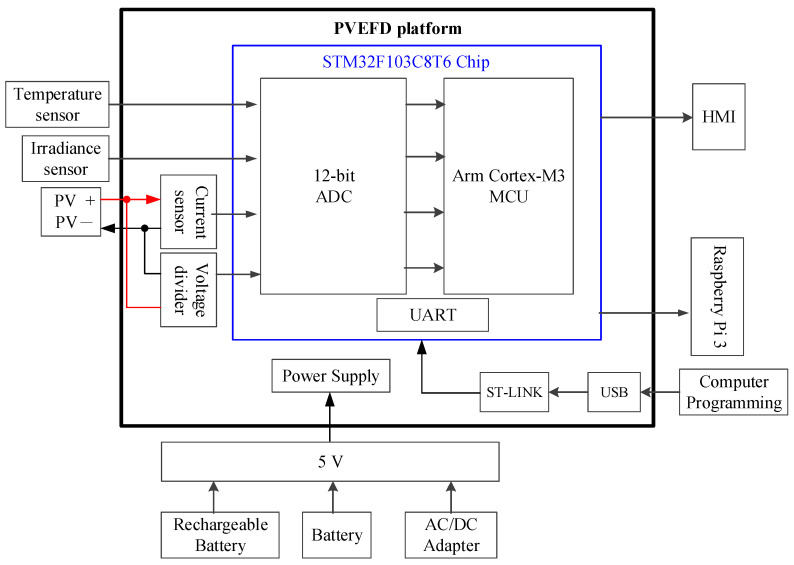
Schematic of proposed IoT-based PVEFD system.

The schematic diagram of the proposed PCEFD system including the STM32F103C8T6 chip with data acquisition configuration is shown in Figure 3. Four types of sensors to measure solar irradiance as well as the back-surface cell temperature, operating voltage, and output current of the PV devices are set up in the experimental test rig. A DAVIS 6450 pyranometer [31] is used to measure solar irradiance with a voltage output signal in the range of 0–3 V_DC_. Five temperature sensors (PT100) are used to measure the cell temperature of the PV module as a voltage signal ranging from 0.2 V_DC_ to 2.8 V_DC_. The output current of the PV module is measured with a current sensor (ACS756) that also has a voltage output signal between 0 and 2 V_DC_ with a sensitivity of 40 mA/V. The specifications of the sensors are tabulated in Table 1.

The *I*-*V* characteristics of PV devices described in Equation (1) reveal the output current is the function of solar irradiance as well as cell temperature, operation voltage, and output current of the PV device; therefore, an iteration must be executed in an algebraic loop. Considering the built-in ADC resolution and computation ability for the HIL simulation of the PV model, the STM32F103C8T6 chip with a built-in Arm cortex-M3 SoC is adopted as a core computing platform featuring cost-effectiveness and computing efficiency. All data of the PV module are directly acquired and forwarded to the 12-bit ADC built in the STM32F103C8T6 chip. The built-in Arm cortex-M3 MCU executes the PV model simulation and calculates the output power based on the sensing data. The instantaneous data can be displayed through HMI. The data are transferred to the Raspberry Pi 3 module through the UART Tx/Rx interface, and then forwarded and saved to the cloud server. An Android mobile app can view the sensing information.

## 3. System Implementation and Experimental Configuration

### 3.1. Implementation of the PVEFD System

**Figure 4:**
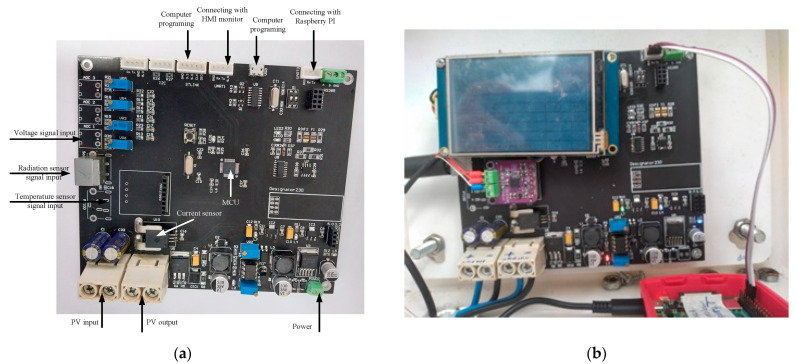
Photographs of proposed IoT-based PVEFD system: (**a**) mainboard; (**b**) connected with peripherals.

The completed PVEFD system is shown in Figure 4. Figure 4a shows the main board consists of the sensing interface, voltage divider, current sensor, an STM32F103C8T6 chip, and peripherals. The device includes a DAQ system for measuring solar irradiance, cell temperature, operating voltage, and PV output current. Additionally, it has an ST-LINK in-circuit debugger and programmer for downloading code from the Arduino IDE to the microcontroller. The Arm Cortex-M3 MCU in the STM32F103C8T6 chip is the most crucial component because it processes signals from the DAQ and calculates the PV output using an internal PV model. Finally, the HMI display shows and compares the measured and simulated results, allowing for testing of the electrical characteristics of commercial PV devices.

### 3.2. Set-Up of Experimental Test Rig

**Figure 5:**
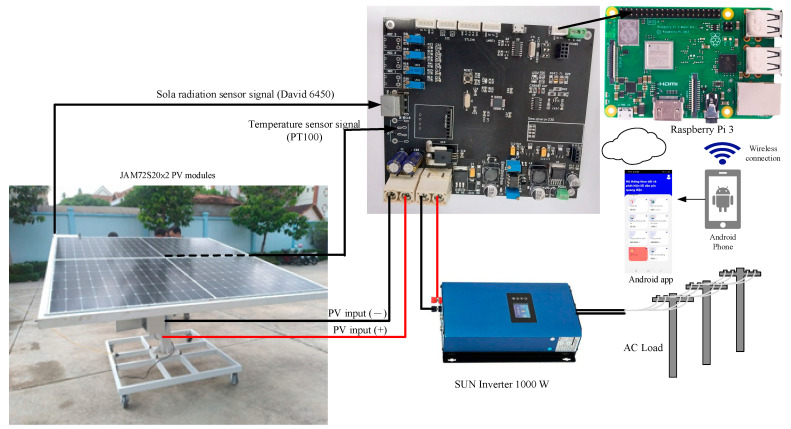
Setup configuration of experimental test rig.

Figure 5 illustrates the experiment test rig, which consists of a 920 W PV system with two JAM72S20-455 PV panels mounted on a rack and a SUN1000 inverter. It was set up at Campus 2 of Lac Hong University in Vietnam. The input parameters for the PV model were taken from the specifications of the commercially available JAM72S20-455 PV module [32], which are listed in Table 2 and configured through a built-in dialog.

## 4. Results and Discussion

### 4.1. Analysis of Experimental Results

**Figure 6:**
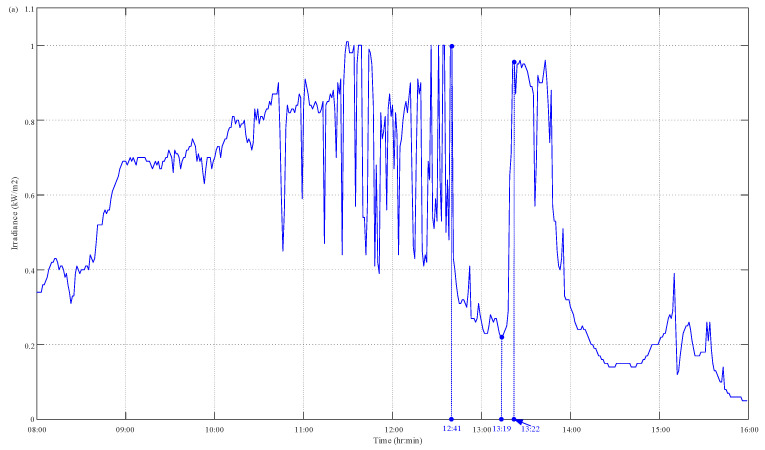

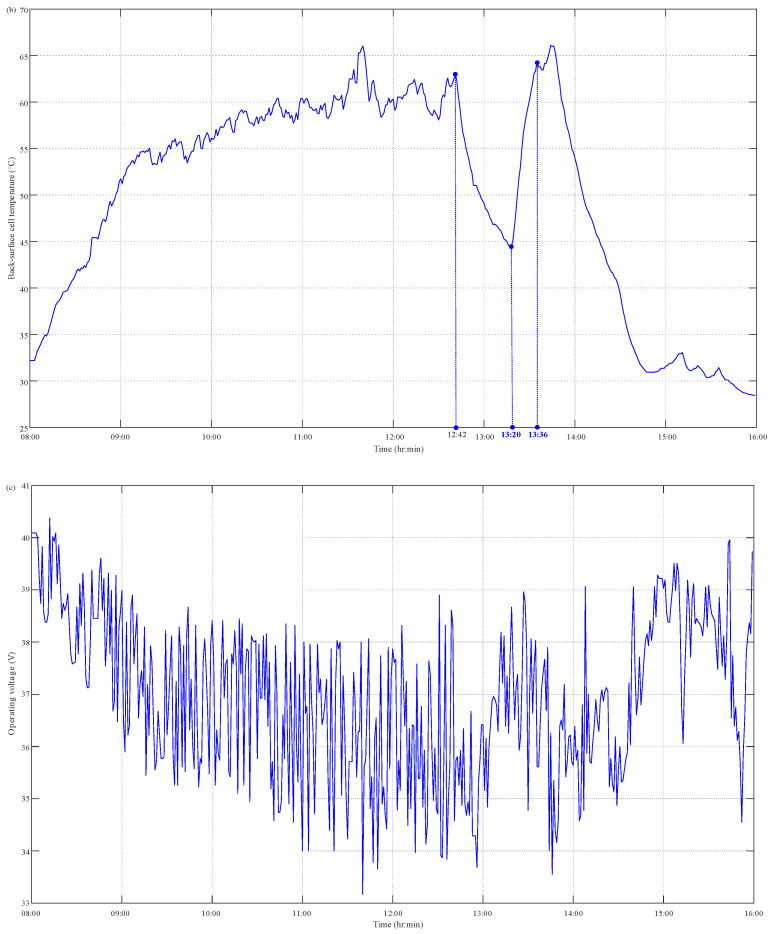
Measured results of PVEFD system: (**a**) irradiance; (**b**) cell temperature; (**c**) voltage.

**Figure 7:**
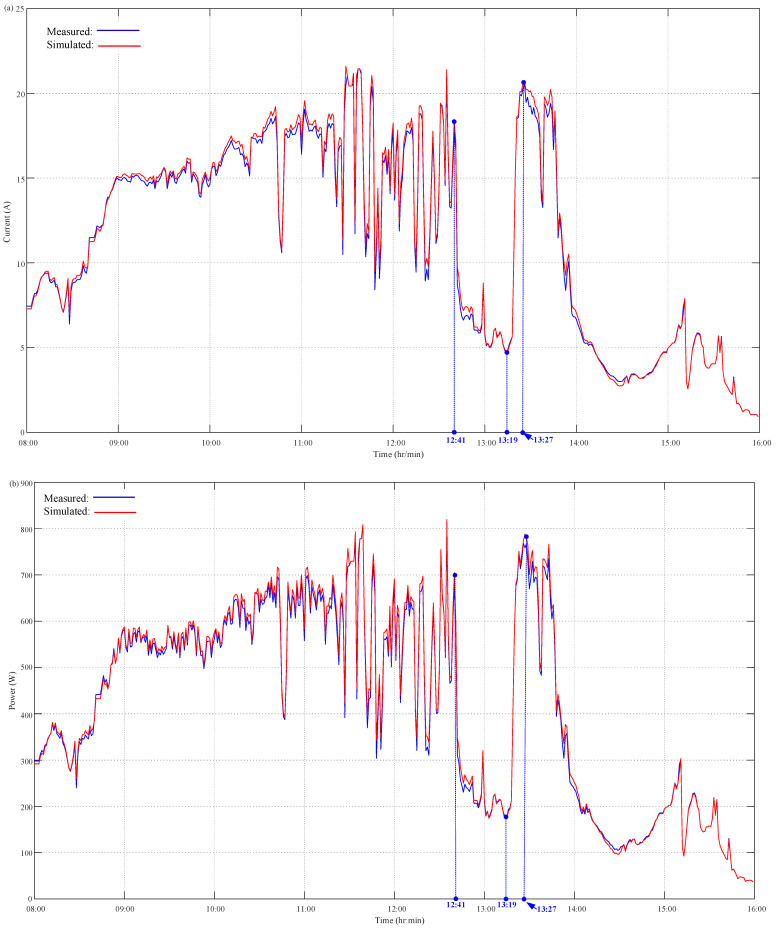
Simulated and measured results of PVEFD system: (**a**) output current; (**b**) output power.

The JAM72S20-455 PV system monitored by the proposed PVEFD system was tested for 7 days consecutively from 11 to 17 November 2022. The PVEFD system measures and stores data on solar irradiance as well as the back-surface cell temperature, operating voltage, and output current of the PV devices. All observations of the solar irradiance as well as the back-surface cell temperatures, operating voltage, and output current of PV devices were recorded at a 1 min interval. Taking a typical sunny day (17 November 2022) for example, the measurement results of all solar radiation, the cell temperature, and the operating voltage of the PV devices are depicted in Figure 6. The simulated data of output current and power can be conducted using a built-in PV model according to the measured data of sunlight intensity, cell temperature, and operating voltage of the PV modules. As highlighted in Figure 6a,b, the solar irradiance decreases at 12:41 and increases at 13:19. The back-surface cell temperature of the PV module behind the solar radiation began to decrease at 12:42 and increased at 13:20. This reveals the solar irradiation has a direct effect on the PV temperature. Furthermore, the solar irradiance continuously rose from 13:19 to 13:22, and the back-surface cell temperature of the PV module increasingly inclined from 13:19 to 13:36. It is an obvious time delay of 14 min. This clearly illustrates the effect of the thermal mass of PV devices on PV temperature interacting with environmental conditions including solar radiation, ambient temperature, and both the speed and the direction of the wind. With the acquired solar irradiance as well as back-surface cell temperature and operating voltage of PV devices, both the output current and power of the PV system were evaluated based on the PVEFD platform through the built-in HIL function; furthermore, the results were compared with the in-field measurements of the experiment. Figure 7 depicts the output current and power for the PV model and real PV devices through HIL simulation and in-field measurement, which significantly presents the simultaneous output current and power of PV devices synchronizing with each other. Furthermore, as referred to in Figure 6a, both output current and power simultaneously changed with the instantaneous variation of solar irradiance as shown at 12:41 and 13:19 in Figure 7a,b. This is because the PV effect can put into effect at much less than 1 s. Moreover, both the output current and power of PV devices reached 20.55 A and 768.66 W, respectively, at the time point of 13:27 as shown in Figure 7a,b. These reveal the combined effect of solar irradiance as well as the cell temperature and operating voltage of PV devices on the PV output current and power. Figure 7a,b present the time-delay effect of the back-surface cell temperature affecting the output current and power of PV devices, which caused a time delay of less than 8 min.

### 4.2. Accuracy Analysis of Experimental Results

**Figure 8:**
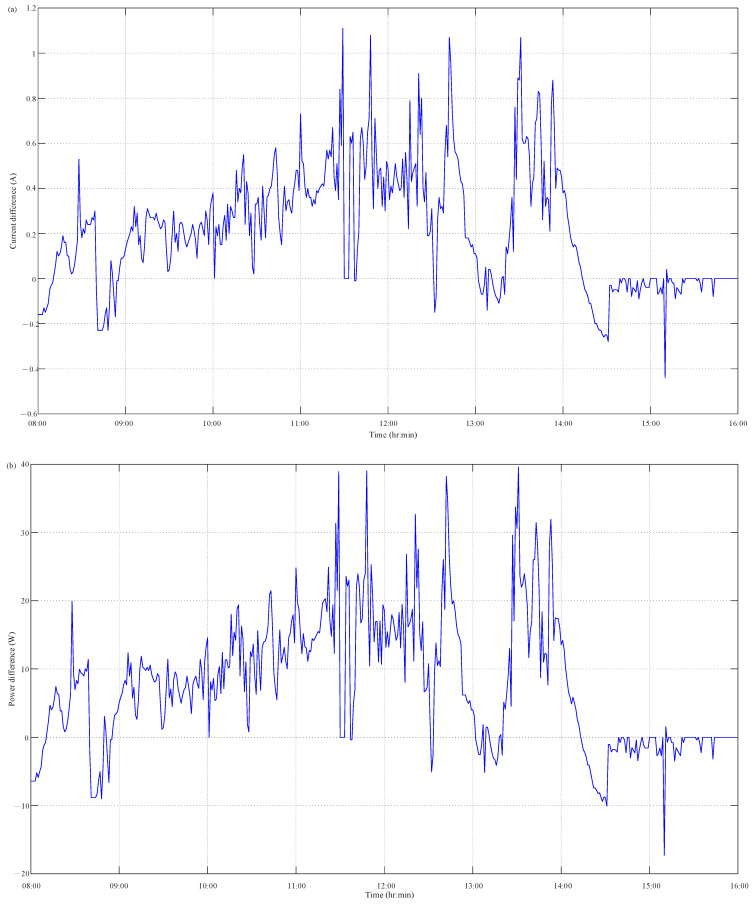
Simulated and measured results of PVEFD system: (**a**) output current; (**b**) output power.

In order to easily assess the accuracy of the proposed PVEFD system, the measurement results of both output current and power of PV devices are taken as reference, and then the difference between the measurement and built-in HIL simulation results of the proposed system is given by

(5) $$e_{j}^{n}=x_{j}^{n}-{\hat{x}}_{j}^{n}$$

where $x_{j}^{n}$ and ${\hat{x}}_{j}^{n}$ are the *j*th measurement values and built-in HIL simulation results, *j* = *I*, *P*_PV_. The performance index of mean absolute error (MAE), mean absolute percentage error (MAPE), and root mean square error (RMSE) and for close-agreement analysis are defined as

(6) $$\mathrm{MAE}=\frac{1}{N}\sum_{n=1}^{N}\left|x_{j}^{n}-{\hat{x}}_{j}^{n}\right|$$

(7) $$\mathrm{MAPE}=\frac{100\%}{N}\sum_{n=1}^{N}\frac{\left|x_{j}^{n}-{\hat{x}}_{j}^{n}\right|}{{\hat{x}}_{j}^{n}}$$

and

(8) $$\mathrm{RMSE}=\sqrt{\frac{1}{N}\sum_{n=1}^{N}{\left(x_{j}^{n}-{\hat{x}}_{j}^{n}\right)}^{2}}$$

where *N* is the observation number. Figure 8 clearly reveals the difference of PV output current and power between measurement and HIL simulation results. Based on the measured and simulated data of PV output current and power, their MAE values of current difference and power difference are found to be 0.2546 A and 9.2999 W, respectively, and their corresponding MAPE values are, respectively, 0.3141% and 1.2156%. Furthermore, the RMSE values of current difference and power difference are found to be 0.3383 A and 12.3099 W, respectively. As compared to the rated current at maximum power point (MPPT) of 10.88 A listed in Table 2, the MAPE value of the PV output current difference for the proposed PVEFD system is much less than 1%. The values of the difference range, MAE, MAPE, and RMSE for the accuracy analysis of PV output current and power are tabulated in Table 3. As compared to the rated current at maximum power point (MPP) of 10.88 A listed in Table 2, the MAPE value of the PV output current difference for the proposed PVEFD system is much less than 1%. This reveals the built-in HIL simulation of the PVEFD system has a close agreement with the in-field current measurement. The cost-effectiveness and sufficient accuracy can be validated. Although, the MAPE value of the PV output power difference for the PVEFD system is greater than 1%, which is mainly caused by the measurement of the PV operating voltage using an MPP tracker (MPPT) in a DC/DC converter or DC/AC inverter, the overall MAPE values of PV output current difference and power difference are much less than 2% with sufficient confidence. The model accuracy of the built-in HIL simulation is significantly related to the measurement accuracy of the pyranometer, temperature, and voltage sensors, which can better the accuracy of the proposed PVEFD system. 

### 4.3. Fault Detection on PV System

**Figure 9:**
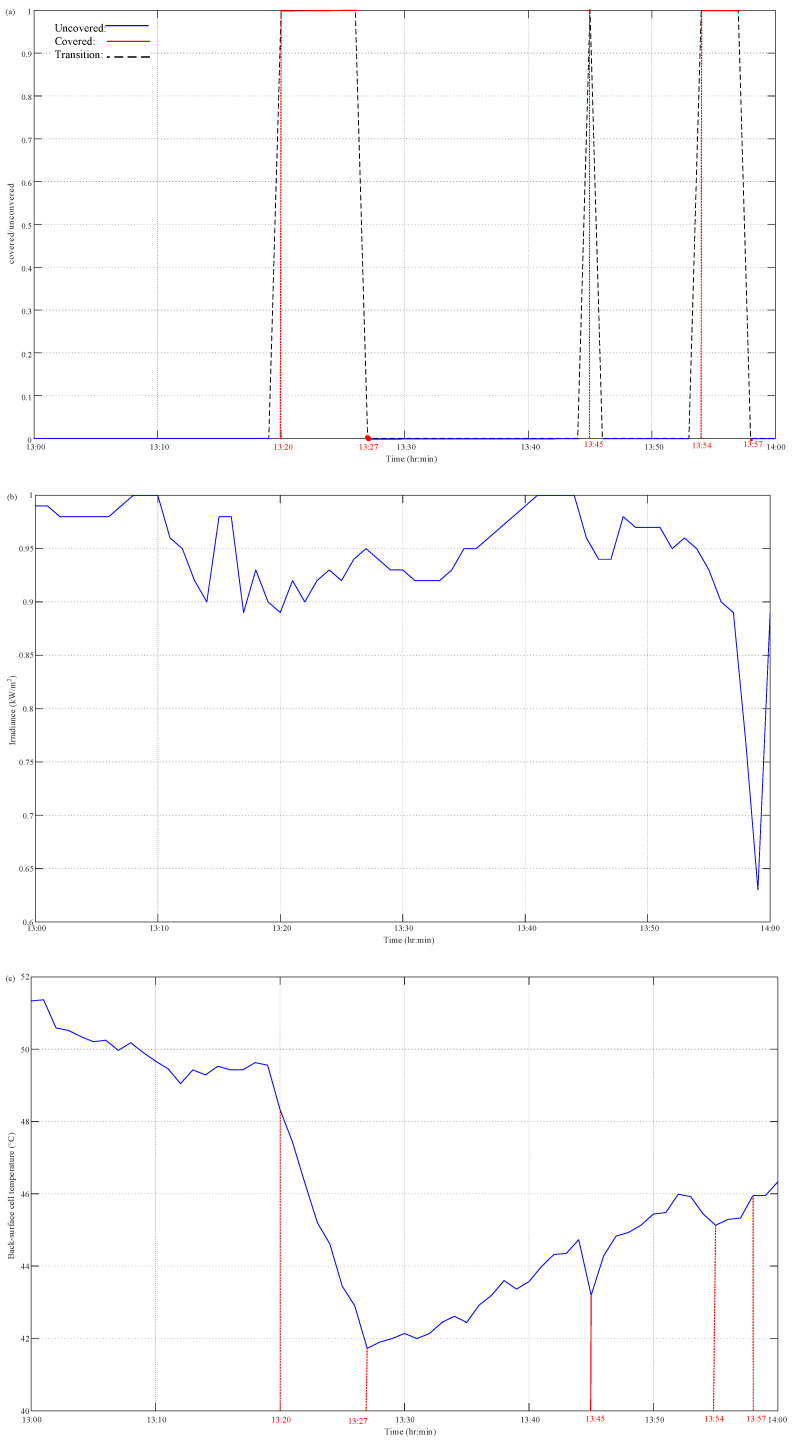

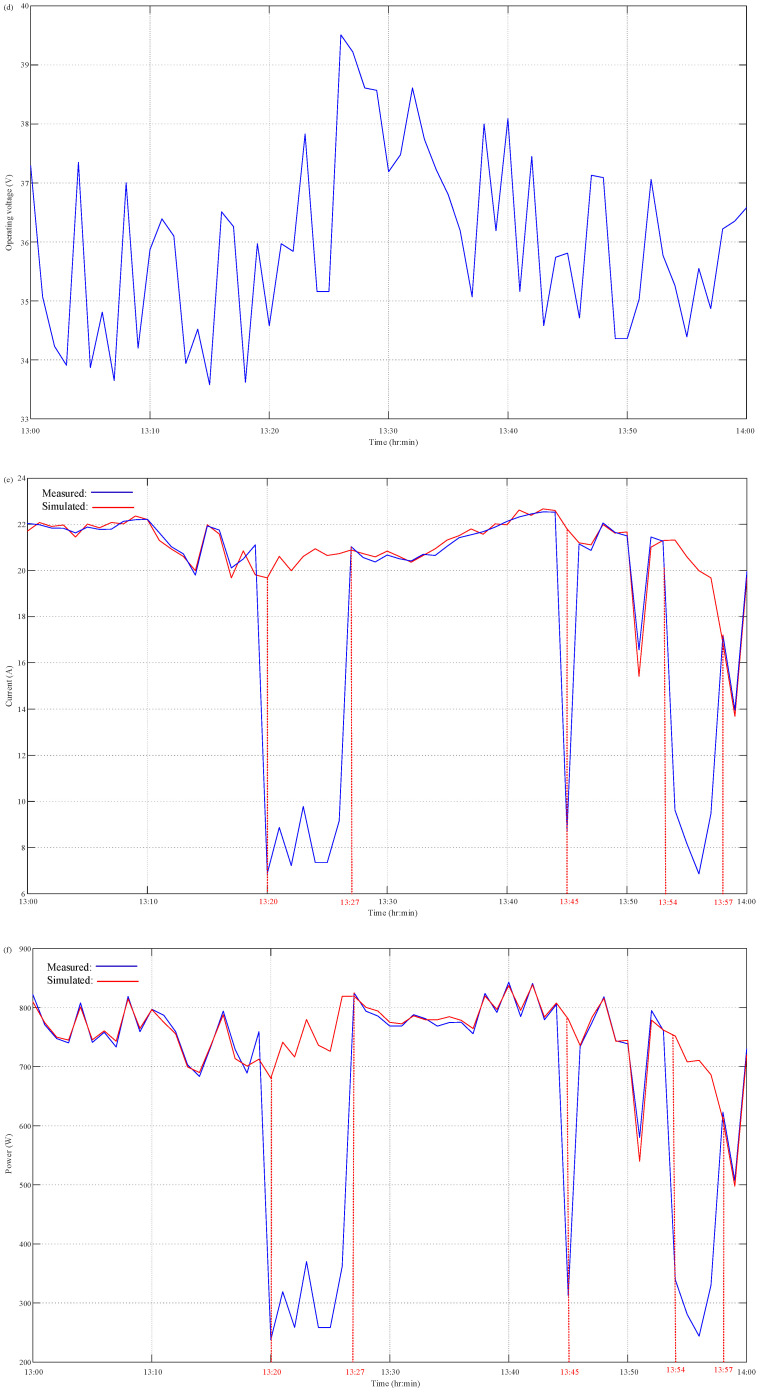
Measured results of PVEFD system on 19 November 2022: (**a**) covered/uncovered operation; (**b**) irradiance; (**c**) cell temperature; (**d**) voltage; (**e**) output current; (**f**) output power.

**Figure 10:**
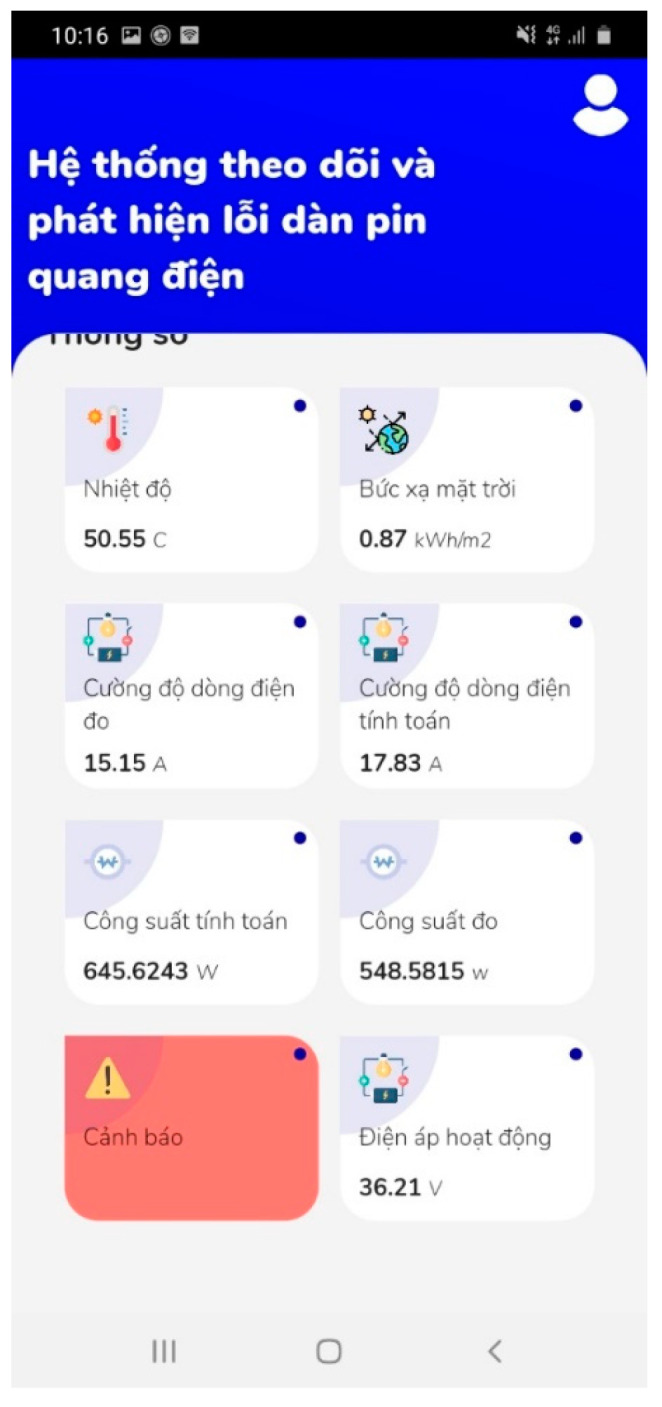
Screenshop of Android app for warning.

**Figure 11:**
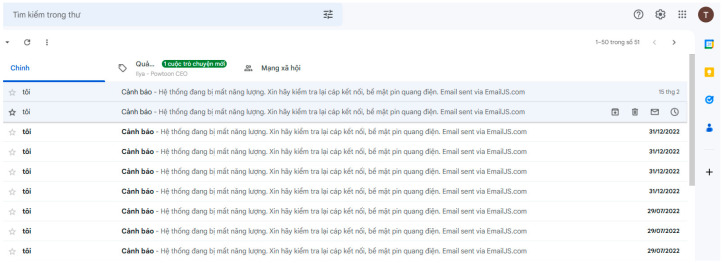
Screenshot of email interface for warning.

The fault caused by partial shading on PV devices and electrical loss that is caused by a mismatch, open circuit, or short circuit of the PV itself can be detected by comparing the measured and HIL-simulated data of the PV output current based on the solar irradiance as well as the back-surface cell temperature and operating voltage of PV devices. Here, a fault type of partial shading effect on PV output current and power was validated. A fault detection test was performed by partially covering a portion of the photovoltaic cell and running the test for 60 min. Figure 9 presents the measurement results of the solar irradiation, cell temperature, and operating voltage of PV devices. Without the consideration of the man-made partial shading effect on measured irradiance, the simulated data of output current and power can be conducted using a built-in PV model according to the measured data of solar irradiation intensity as well as cell temperature and operating voltage of PV modules. The measured irradiance and the model closely resemble the module’s current output, as seen in Figure 9. As shown in Figure 9a, the JAM72S20-455 PV panel was covered or uncovered with a piece of low-transparency paper for a few minutes and then uncovered from 13:00 to 14:00 on 19 November 2022. There were three covered/un-covered operations at 13:20, 13:45, and 13:54 with 7 min, 1 min, and 3 min time durations, respectively. The measurement of the solar irradiation as well as the back-surface cell temperature, operating voltage, output current, and power of the PV devices by the proposed PVEFD system with a time interval of 1 min is also depicted in Figure 9b–f. In addition, the results of the HIL simulation of PV output current and power in the proposed PVEFD system are depicted in Figure 9e,f. As highlighted in Figure 9c, the back-surface cell temperature decreased at 13:20, 13:45, and 13:54, and gradually increasing after uncovering the paper, pointing to the direct influence and time-delay effect of solar radiation on the PV temperature because of the thermodynamic behavior of PV devices interacting with the ambient environment as discussed above. Figure 9e,f show both PV output current and power significantly decreased, posted to the shading effect of low-transparency paper; however, those of the HIL simulation still based on the acquired solar irradiance as well as the back-surface cell temperature and operating voltage of the PV devices without taking the blackout effect into consideration. The slump of PV output current or power caused by the unwilling shading can be used for fault detection and the warning function. Furthermore, the evaluation and fault detection app for Android devices collects data from hardware with embedded measurement and fault detection algorithms and sends it to a server. The app features an interface, and the screenshot is presented in Figure 10. The warning signal is not only displayed on the Android mobile app but also sent to the pre-installed email. Figure 11 presents the email interface for the warning.

### 4.4. Discussions

As illustrated in Section 4.1, the ability to evaluate the on-site performance of PV devices for the proposed PVEFD system has been validated by comparing the measured output current and power of PV systems with those of the built-in HIL simulation results based on in situ measurement of solar irradiance as well as the back-surface cell temperature, operating voltage, and output current of PV devices. Figure 7 clearly reveals the simultaneous measured and simulated output current and power of PV systems, respectively. Taking the measured output current and power of PV devices as a reference, the difference analysis of PV current and power between the HIL simulation and measurement results can show that the MAPE values of the difference for PV current and power are 0.3141% and 1.2156%, respectively. This further confirms the proposed PVEFD system has sufficient accuracy for on-site measurement. Based on the results of the HIL simulation by the acquired solar irradiance as well as the cell temperature and operating voltage of the PV devices, the partial shading effect on PV output current and power can be detected and an online alert can warn the stakeholders in time. 

Based on the comparison of the difference between the measurement and HIL simulation results of the PV output current and power, the mismatch fault due to partial shading effect or PV aging as well as other electrical faults such as open-circuit faults and short-circuit faults can be detected, and an alert can be given, automatically. Table 4 tabulates the different functionalities of the published works for the fault detection of PV devices through experimental validation.

## 5. Conclusions

This paper presents a photovoltaic (PV) evaluation and fault detection (PVEFD) system based on the Internet of Things (IoT) architecture, which uses the STM32F103C8T6 chip and Raspberry Pi model 3. A built-in PV mathematical model for commercialized PV modules is used for the hard-in-the-loop (HIL) simulation for PV performance evaluation and fault detection function. The proposed PVEFD system has been experimentally validated in real operating conditions, and both measured and simulated data can be forwarded to the cloud server through a WiFi network. Results reveal that the proposed PVEFD system is accurate and reliable, making it a valuable tool for PV system engineering. Specifically, the PVEFD system offers several advantages such as low cost, easy setup, and the ability to quickly detect faults in PV systems. The system also enables the ability of easy tracking of PV power systems through an Android app and provides early warning in case of power loss. Additionally, the proposed PVEFD system can achieve cost-effectiveness through its compact configuration and sufficient precision, making it an attractive option for data acquisition and performance evaluation in commercial PV devices under in-field operations.

## Figures and Tables

**Table 1:** Specifications of sensor devices.

Sensor Types	Characteristics	Specifications
DAVIS 6450 (Pyranometer)	Power supply	3 V
	Sensitivity	1.67 mV/1 W/m^2^
	Accuracy	±5%
	Range	0 to 1800 W/m^2^
	Resolution and units	1 W/m^2^
ACS756SCB-050B (Current sensor)	Power supply	3–5 V
	Sensitivity	40 mV/A
	Accuracy	±5%
	Current sensing	50 A
PT100 (Temperature sensor)	Temperature range	−50 °C to +200 °C
	Accuracy	±1 °C
	Nominal resistance	100 Ω at 0 °C

**Table 2:** Specifications of JAM72S20-455 PV module [30].

Characteristics	Specifications
Rated Maximum Power (*P*_max_)	455 W
Open-Circuit Voltage (*V*_OC_)	49.85 V
Maximum Power Voltage (*V*_mpp_)	41.82 V
Short-Circuit Current (*I*_SC_)	11.41 A
Maximum Power Current (*I*_mp_)	10.88 A
Module Efficiency	20.4%
Power Tolerance	0–5 W
Temperature Coefficient of *I*_SC_ (α_ *I*_SC_)	+0.044%/°C
Temperature Coefficient of *V*_OC_ (β_ *V*_OC_)	−0.272%/°C
Temperature Coefficient of *P*_max_ (γ_ *P*_max_)	−0.350%/°C
Cell type	Mono-Crystalline
Number of cells	144

**Table 3:** Accuracy analysis of PVEFD system.

Items	Difference Range	MAE	MAPE	RMSE
Current	−0.4400–1.1100 A	0.2546 A	0.3134%	0.3384 A
Power	−17.2964–39.5900 W	9.2999 W	1.2156%	12.3099 W

**Table 4:** Comparison of fault detection function for PV devices.

No.	System Function			Reference
	Detection	Localization	Categorization	
1	✓	×	Array aging, shadow, and short-circuit/open-circuit fault	Xie et al. [25]
2	✓	✓	PV faulty in a string, single or multiple faulty PV strings, partial shading, and soiling on a string.	Iqbal et al. [26]
3	✓	×	Short-circuit/open-circuit fault, array aging, shadow, mismatch fault, and unidentifiable fault	Voutsinas et al. [27]
4	✓	✓	Line-to-line (L–L) and line-to-ground electrical faults	Zakir et al. [29]
5	✓	×	Partial shading fault, PV aging, short-circuit/open-circuit faults	Proposed PCEFD system

## Data Availability

Not applicable.
